# Evaluating the effectiveness of behavior change techniques in health-related behavior: a scoping review of methods used

**DOI:** 10.1093/tbm/ibx019

**Published:** 2018-02-10

**Authors:** Susan Michie, Robert West, Kate Sheals, Cristina A Godinho

**Affiliations:** 1 Department of Clinical, Educational and Health Psychology, Centre for Behaviour Change, University College London, UK; 2 Department of Behavioural Science and Health, Cancer Research UK Health Behaviour Research Centre, University College London, UK

**Keywords:** Behavior change techniques, Evidence synthesis, Behavioral interventions, Behavior, Ontology

## Abstract

Behavior change interventions typically contain multiple potentially active components: behavior change techniques (BCTs). Identifying which specific BCTs or BCT combinations have the potential to be effective for a given behavior in a given context presents a major challenge. The aim of this study was to review the methods that have been used to identify effective BCTs for given behaviors in given contexts and evaluate their strengths and limitations. A scoping review was conducted of studies that had sought to identify effective BCTs. Articles referring to “behavio(u)r change technique(s)” in the abstract/text were located, and ones that involved identification of effective BCTs were selected. The methods reported were coded. The methods were analyzed in general terms using “PASS” criteria: Practicability (facility to apply the method appropriately), Applicability (facility to generalize from findings to contexts and populations of interest), Sensitivity (facility to identify effective BCTs), and Specificity (facility to rule out ineffective BCTs). A sample of 10% of the studies reviewed was then evaluated using these criteria to assess how far the strengths and limitations identified in principle were borne out in practice. One hundred and thirty-five studies were identified. The methods used in those studies were experimental manipulation of BCTs, observational studies comparing outcomes in the presence or absence of BCTs, meta-analyses of BCT comparisons, meta-regressions evaluating effect sizes with and without specific BCTs, reviews of BCTs found in effective interventions, and meta-classification and regression trees. The limitations of each method meant that only weak conclusions could be drawn regarding the effectiveness of specific BCTs or BCT combinations. Methods for identifying effective BCTs linked to target behavior and context all have important inherent limitations. A strategy needs to be developed that can systematically combine the strengths of the different methods and that can link these constructs in an ontology of behavior change interventions.

## Teaser

Methods for identifying effective behavior change techniques for given behaviours and contexts all have important limitations. We need a way to combine methods to arrive at reliable effectiveness estimates.

**Implications**

**Practice:** When deciding what combination of BCTs to use in an intervention, assessment of likely effectiveness needs to be based on integration of findings across different methods; until a formal method for doing this is developed, conclusions need to be subject to major qualifications.

**Policy**: When considering components to include in behavior change strategies, policymakers need to combine evidence from the full range of methods available and make conclusions subject to major caveats; given this uncertainty, monitoring outcomes and adjusting policies in the light of experience are crucial.

**Research**: There is an urgent need to develop formal methods for combining evidence from different types of evaluation to arrive at judgments concerning the likely effect sizes of BCT combinations tailored to target behavior and context; this could benefit from organizing evidence using an “ontology” of behavior change interventions.

## INTRODUCTION

The primary practical purpose of research into behavior change is the development of interventions that will be effective, subject to other constraints such as affordability [1, 2]. In doing so, one wants to be able to draw on research findings that identify behavior change techniques (BCTs) that, if enacted appropriately, are most likely to effect the desired change. This will depend not only on the behavioral outcome but also on the ways the BCTs are delivered and the context. This paper reviews the methods that researchers have used to identify relevant BCTs for use in behavior change interventions and analyses their strengths and limitations specifically for this purpose.

When characterizing the potentially active ingredients of a behavior change intervention, a distinction can be made between the “content” of interventions (their putative active components) and the way in which they are delivered. Content can be characterized in terms of BCTs [3–7], defined as the smallest identifiable components that in themselves have the potential to change behavior [8]. BCT taxonomies have been developed that provide a standardized method of classifying intervention content [9]. In Michie et al.’s [3, 9] taxonomy of BCTs, 93 BCTs, in 16 groupings, were distinguished addressing the different potentially important targets of capability, opportunity, and/or motivation [1, 2]. A key task in behavioral science can be seen as understanding the extent to which BCTs contribute to the effectiveness of interventions of which they form a part.

In health care, behavior change interventions are aimed at a range of behavioral outcomes: preventing and stopping people engaging in harmful or risky behaviors (e.g., smoking), promoting engagement with health protective behaviors (e.g., exercising or engaging with cancer screening programs), switching from more harmful to less harmful forms of a behavior (e.g., reducing excessive drinking or excessive speed while driving), promoting effective use of health care interventions (e.g., improving medication adherence), and promoting effective self-management of diseases (e.g., monitoring blood glucose concentrations). Interventions aimed at changing health professional behaviors can involve the following: ensuring that those working in health care follow evidence-based guidelines (e.g., in reducing antibiotic prescribing, improving hand hygiene) or improving the way that they follow procedures (e.g., when administering drugs or making diagnoses). In all of these cases, the theory and practice of behavior change can be improved by conducting interventions and assessing their effects. Specifying the content of behavior change interventions in terms of BCTs enables the identification of potentially effective components within complex interventions, both in primary research (e.g., [10–12]) and in evidence syntheses in systematic literature reviews (e.g., [13–16]).

Factors that complicate the process of identifying effective specific BCTs or BCT combinations include the following: (a) the effect of a single BCT may be very small, (b) many BCTs typically occur together in a given intervention, (c) BCTs may interact with each other to amplify or reduce effectiveness, (d) effectiveness of BCT depend on how they are delivered, (e) effectiveness may depend on specific features that are not captured by the BCT classification being used, and (f) all the preceding may vary across context (population and setting). Complicating matters further, methods of identifying effective BCTs that involve synthesizing findings across studies depend critically on accurate and complete descriptions of interventions, the target populations and settings, and use of comparable behavioral outcomes.

Despite this, there is evidence that the task is tractable. For example, it has been possible to identify BCTs involved in behavioral support for smoking cessation that have been associated with higher success rates of local stop smoking services in England [17]. This has formed the basis for guidance on service provision and learning objectives in training courses, and the use of this guidance and training has been found to be associated with increased success rates [18]. Progress in behavioral science and its application depends on the success of this kind of exercise. Without it, we cannot build generalizable knowledge or create new interventions that have a high likelihood of success. There have also been developments in linking the key constructs of behavior change interventions (BCTs, their mode of delivery, mechanisms of action, target behavior and context) into what is known as an ontology of behavior change interventions [19]. Linking constructs into one knowledge structure recognizes that BCTs may be differentially effective according to their mode of delivery, type of target behavior and context.

An informal assessment of the research literature shows that methods that are used to establish effectiveness of complete behavior change interventions are also often used to identify effective BCTs. In terms of primary studies, these include randomized controlled trials (RCTs) and comparative observational studies. In terms of evidence synthesis, they include meta-analyses. Other methods are also used such as meta-regressions. How far these methods can be successfully applied to identifying effective BCTs is not clear, however. We set out to establish what methods had been used and to analyze their strengths and limitations. The task is different from that of identifying effective interventions for the reasons given above. The task is essentially an identification problem in a complex environment with limited resources. We can therefore draw on concepts from signal detection theory [20] and applied research methodology [21] for criteria to use in evaluating the methods. We propose that these can be distilled to what may be termed the “PASS” criteria:

1 Practicability: How well can the method achieve the desired objectives within available time and resource constraints?
2. Applicability: How well does the method allow generalization to populations and settings of interest?
3. Sensitivity: How well suited is the method to picking up potentially effective BCTs for a given behavior and context if these are present?
4. Specificity: How well does the method identify BCTs that will not be effective for a given behavior and context?

This paper aimed to review methods used to identify effective BCTs or BCT combinations and analyze their strengths and limitations according to the above criteria.

## METHOD

### Identification of articles

**Fig. 1:**
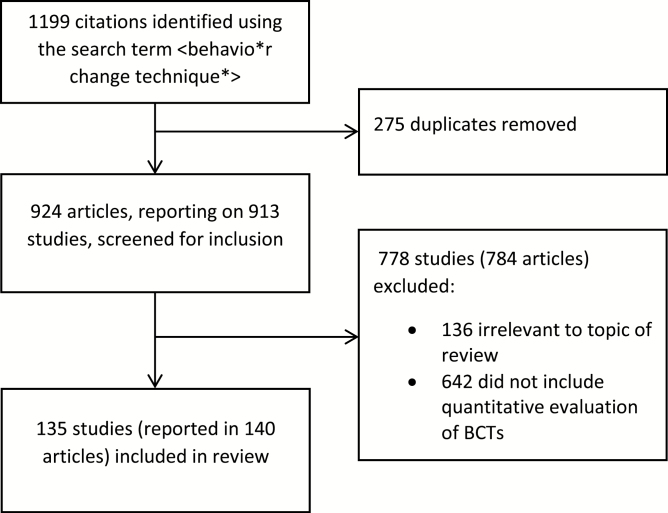
Flow chart for identification of studies

We searched the electronic databases Web of Science, PubMed, and PsycInfo using the search term <behavio*r change technique*> up to end of February 2015. Titles and abstracts were screened, and articles were selected for full-text analysis if they were written in English and contained a quantitative evaluation of the effectiveness of individual BCTs or specific BCT combinations within interventions aimed at changing health behavior. The process involved initial screening by K.S. and C.G., following which articles were identified and selected in consultation with S.M. and R.W. See Fig. 1 for a flowchart showing the process of study identification. The goal was not to find every BCT evaluation that had been conducted but to have reasonable confidence that the different methods used had been canvassed and to have a general indication as to their relative frequency of use.

### Coding of methods for identifying effective BCTs

An initial coding frame was developed, informed by the BCT evaluation methods identified in the literature reviewed by the UK’s National Institute for Health and Social Care Excellence (NICE) in developing its guidance on behavior change [22]. Evaluation methods coded were as follows: experimental manipulation of BCTs including RCTs, observational studies comparing interventions with or without targeted BCTs, meta-analyses of comparisons of BCTs, meta-regressions assessing relative effect sizes of interventions with or without specific BCTs, and characterizing effective interventions in terms of their BCTs. An additional category was identified and added during the course of the current review: meta-classification and regression trees (CART). Studies were each assigned to one evaluation method category by K.S. and C.G. in consultation with S.M. and R.W.

### Analysis of the strengths and limitations of the methods

The authors first engaged in a process of identifying in broad terms the strengths and limitations of the methods found in the review, specifically for the task of identifying potentially effective BCTs for particular behaviors in particular contexts, using the PASS criteria set out earlier. The process of analyzing strengths and limitations of the methods involved complex judgments, combining a range of factors. The PASS criteria were designed to provide a framework for judgments about strengths and limitations, but a degree of subjectivity was unavoidable. A 10% of the studies were selected for detailed analysis to assess how far these strengths and limitations were manifest in that study. Each study was given a rating on sensitivity, specificity, and applicability from 1 to 3 by a researcher (HKU) in terms of how satisfactory the method had proved (1 = unsatisfactory, 2 = marginally satisfactory, 3 = satisfactory). The study was also rated from 1 to 3 in terms of practicability of replication bearing in mind the time and resources required (1 = impracticable, 2 = marginally practicable, 3 = practicable). The results were checked by R.W., and minor modifications were made.

## RESULTS

### Evaluation methods used

Of the 913 studies identified by our search, 135 met our inclusion criteria, reported in 140 articles. Of the excluded studies, 136 were irrelevant to the topic of this review, and 642 were relevant to behavior change but did not include an assessment of the effectiveness of BCTs. The latter included 24 review articles that had used a taxonomy of BCTs to code intervention content regardless of effectiveness and 37 study protocols or intervention development articles.

Table 1 shows the methods found in the review to investigate effectiveness and the number of studies that used each method. References to studies categorized as using each method can be found in the online Supplementary Material. As might be expected, the most commonly used method was the use of individual empirical studies. However, studies characterizing what had been found to be effective interventions in terms of their component BCTs, meta-analyses, and meta-regressions were also common. Comparative observational studies were rare and only one example was found of use of meta-CART.

**Table 1:** Methods used to evaluate effectiveness of behavior change techniques (BCTs)

Evaluation method	What it involves	Strengths and limitations using PASS criteria	Total number of references included in the review (Supplementary File)
Experiments (including RCTs); For example, providing feedback on expired air carbon monoxide concentrations to aid smoking cessation (Shahab et al., 2011).	Adding or removing one or more BCTs under experimenter control and looking for differences in effectiveness.	P: Only feasible for evaluating small numbers of BCTs at any one time; resources required for adequately powered studies can be prohibitive; ethical and pragmatic barriers are often insuperable; timescales tend to be long (usually 3 or more years for experiments involving important outcomes). A: Generalization beyond the study population and setting is often problematic, particularly where informed consent is required and/or recruitment is low. Se: Where effects are found, can provide confidence in attributing these to BCTs, but this is still problematic when there is a loss to follow-up, differential uptake of the intervention, or potential bias in the measurement of outcomes. Sp: Inability to detect effects may be due to a wide range of factors other than ineffective BCT(s), including low power, inadequate delivery of the BCT(s), and low measurement accuracy.	73
Comparative observational studies; For example, identification of BCTs associated with higher success rates of stop smoking services in England (West et al., 2010).	Using naturally occurring variation in clinical or public health practice in inclusion of BCTs and outcomes to identify associations between BCT inclusion and intervention effectiveness.	P: Can be very cost effective if data are already available or can be recorded as part of routine care; completeness and accuracy of data collection are often low; rely on naturally occurring variation in use of BCTs; fidelity of delivery of BCTs may be low or unknown. A: Can involve “real-world” settings and populations making generalization less problematic. Se: Can make use of very large data sets increasing potential sensitivity; number of permutations of BCTs may undermine detection of interactions; susceptible to high bias and error of outcome measurement. Sp: Causality has to be inferred (usually by statistical adjustment for potential confounding variables such as mode of delivery, setting, population, and other BCTs); can result in high false positive rate where multiple BCTs are being considered.	4
Meta-analyses of experimental studies; For example, implementation intentions as actions plans to promote behavior change (Gollwitzer & Sheeran, 2006).	Statistically pooling the results or two or more experiments evaluating one or more BCTs as above.	P: Can be conducted within a few months at relatively low cost compared with empirical studies; often there are too few studies that are sufficiently similar in terms of interventions and methodology; studies mostly involve testing packages of BCTs. A: Can provide confidence about generalizability across specific contexts; generalization of findings is still constrained by contextual factors of the studies included. Se: When there are enough high-quality studies, they can provide a high level of confidence in effectiveness of BCTs and provide robust effect size estimates. Sp: Can be biased by failure of researchers to report negative findings; fidelity of delivery of BCTs may be low or unknown or variable.	16
Meta-regressions; For example, identification of self-monitoring, goal setting, and actions plan as effective BCTs in promoting physical activity and healthy eating (Michie et al., 2009).	Identifying inclusion versus exclusion of BCTs or their combinations as moderators of effect sizes in meta-analyses of multi-component interventions.	P: Can be conducted within a few months at relatively low cost compared with empirical studies; often there are too few studies that are sufficiently similar in terms of interventions and methodology; studies mostly involve testing packages of BCTs; interventions and controls are often not described well enough to be able to identify BCTs and important contextual factors. A: Can provide confidence about generalizability across specific contexts; generalization of findings is still constrained by contextual factors of the studies included. Se: May detect effects that are too small to be picked up in individual studies; rely on large number of studies that vary in use of BCTs in intervention and control condition; apparent BCT effects may be due to other study features. Sp: Failure to detect effects may be due to a large number of factors in the contributing studies too little variation in BCT use across studies.	9
Meta-CART (Classification and Regression Trees) (Dusseldorp et al., 2014).	A set of computational learning methods that produce either “classification” or “regression” trees, depending on whether the dependent variable is categorical or numeric, respectively. Starting with a “root” node, the sample is partitioned successively to create a branching tree of nodes with each branch terminating in a “leaf”, which is the subsample that differs maximally from other subsamples on the dependent variable.	P: Can be conducted within a few months at relatively low cost compared with empirical studies; often there are too few studies that are sufficiently similar in terms of interventions and methodology; studies mostly involve testing packages of BCTs; interventions and controls are often not described well enough to be able to identify BCTs and important contextual factors. A: Can provide confidence about generalizability across specific contexts; generalization of findings is still constrained by contextual factors of the studies included. Se: Well suited for testing BCT interactions; susceptible to false positives; apparent BCT effects may be due to other study features. Sp: Only able to detect a small proportion of BCT interactions that might be effective without extremely large numbers of studies.	1
Characterizing effective interventions; For example, identifying BCTs included in effective behavioral support interventions for smoking cessation (Michie, Churchill & West, 2011).	Identifying BCTs included in interventions found to be effective in RCTs May vary in implementation from inclusion of BCTs that are present in at least one effective intervention to those that have been present in all effective interventions.	P: Relatively inexpensive and can be undertaken in a few months; relies on accurate characterization of BCTs in intervention conditions. A: Can provide confidence about generalizability across specific contexts; generalization of findings is still constrained by contextual factors of the studies included. Se: High probability of picking up BCTs that are effective among those that are frequently tested but unable to differentiate ones that are less effective from those that are tested less often. Sp: Likely to include BCTs that are ineffective but included as part of intervention packages.	32

*BCTs* behavior change techniques; *RCTs* randomized controlled trials; *P* Practicability; *A* Applicability; *Se* Sensitivity; *Sp* Specificity.

### Strengths and limitations of evaluation methods used

Table 1 summarizes an analysis of strengths and limitations of the different methods using the steps described above.

Individual experimental studies can provide the strongest indication of causal relationship between specific BCTs or BCT combinations and effect sizes in a given set of circumstances, at least when there are no biasing factors such as loss to follow-up. However, this approach has major limitations. It is not feasible when comparing large numbers of individual BCTs or their combinations. Expected effect sizes of individual BCTs are usually small, so large sample sizes are required for adequate power and, even with factorial or fractionated factorial designs, the resources needed to undertake such experiments are typically prohibitive [23, 24]. Generalization beyond the specific circumstance is likely to be limited. Effectiveness is influenced by implementation fidelity, which is often low [25, 26]. Ethical and practical considerations often preclude random allocation of participants to experimental and comparison conditions [25, 27].

By examining naturally occurring covariation, comparative observational studies avoid many of the problems with experimental studies, such as ethical and practical constraints in randomly allocating participants to conditions. Because they do not involve setting up interventions, but rather record inputs, processes and outcomes for interventions that are already in existence are typically much less expensive to run. Where there is naturally occurring variation within very large regional or national programs, they can involve large number of participants and thus have power to detect small effect sizes [17, 28]. They typically involve interventions that are being delivered in the real world where fidelity is likely to be lower than in experimental studies. Their key limitation is in the confidence with which effects can be ascribed to BCTs. This can be addressed to some degree by statistical adjustment for potential confounding variables and establishing that factors that determine application of different BCTs to different members of the target group are not likely to be confounded with outcome. In addition, the quality of data is often lower than in experimental studies, with large amounts of missing data and measures that are poorly applied [29]. This can add both to random error and bias.

Meta-analyses of experimental studies can provide generalizable conclusions with a high degree of confidence in causal inference but are limited by all the factors that limit the studies that contribute to them and several others in addition [30, 31]. Firstly, the approach is limited by the viability of assumptions underpinning the aggregation of data from those studies. For example, if a category of target behavior is chosen that is heterogeneous with regard to effectiveness of BCTs, it will produce misleading results. Equally important, a given category of BCT may be implemented very differently in one study versus another. Secondly, the approach is limited by the quality of the studies contributing to the meta-analyses and the difficulties in taking account of variations in methodological quality. The common approach of assigning a quality score and weighting studies accordingly can fail to remedy this because specific deficiencies can fatally undermine a study while only reducing its score by a certain amount. Thirdly, the approach is limited by the small number of relevant experimental studies [32].

Meta-regressions allow pooling of data from experimental studies to draw conclusions about associations between BCTs included in interventions and effect size. Their main strength is in the ability to aggregate data over many different studies to find patterns of association [16, 33]. A major limitation is that when comparing effect sizes across studies, one is moving from an experimental to a correlational design. This means that there may be unmeasured confounding that accounts for associations observed (such as variations in combinations of BCTs, mode of delivery, population and setting) [34]. Another important limitation is that meta-regressions are reliant on descriptions of the key variables. Yet, there is good evidence that published descriptions of intervention and control conditions are very incomplete [34, 35]. A third limitation is that one requires a large number of studies and sufficient variation among the studies in terms of intervention components and effect sizes in order to have a chance of detecting relevant associations [36]. When it comes to assessing the effect of combinations of BCTs, it is imperative to identify in advance what combinations would be expected to yield what effects. This requires use of sound theoretical principles to avoid arriving at misleading conclusions [14, 37]. The most commonly used evaluation method is to identify BCTs present in interventions found to be effective in RCTs. This has the merit of providing a basis for developing intervention manuals or prescriptions in the future, where the intervention package can be assumed to yield an effect similar to what has been achieved in RCTs. However, it also runs the risk of including BCTs that do not add to effectiveness but happen to be included in effective interventions. A second limitation is that it does not permit relative effectiveness of BCTs to be assessed. Thirdly, it does not provide a basis for evaluating different BCT combinations.

Meta-CART has the potential to evaluate circumstance-sensitive effectiveness of BCTs and BCT combinations [38]. It uses what is essentially a correlational design similar to meta-regression but searches for regressions in particular substrata of the data set of interest, recognizing that some BCTs or BCT combinations may be effective in some circumstances but not in others. The obvious strength of this approach is that it allows for heterogeneity of BCT effectiveness. The major limitation is that it requires very large samples and many studies varying in important ways to be able to detect these effects reliably. It will be very rare that there are sufficient studies for associations to meet standard criteria of statistical significance.

Characterizing interventions found to be effective in terms of their BCTs have the key advantage that it is likely to include BCTs that have the potential to be effective but suffers from the key limitation that it may also include large numbers of BCTs that do not contribute to effectiveness but are frequently included in intervention packages.

A sample of 10% (*n* = 13) of the studies was evaluated using the PASS criteria. Table 2 is an example that shows the PASS analysis results of the studies evaluated in the review. It is important to note that the ratings were not judgments of the quality of the studies but of the extent to which the studies would allow confident conclusions about the effectiveness or otherwise of specific BCTs or BCT combinations. This analysis produced results that were consistent with the more general analysis of the strengths and limitations of the different methods. Only weak conclusions about BCT effectiveness could be drawn from the studies.

**Table 2:** PASS analysis of 13 studies in the review

Reference in Supplementary File	Study method	Practicability (1–3)	Applicability (1–3)	Sensitivity (1–3)	Specificity (1–3)	Justification
Reference in Supplementary File	Study method	Practicability (1–3)	Applicability (1–3)	Sensitivity (1–3)	Specificity (1–3)	Justification
Ref no. 47	Exp	1	1	1	1	P: Difficult to recruit participants; full scale trial would be difficult to achieve. A: Difficult to generalize to population of interest in settings that could deliver the intervention. Se: Small sample limits detection of effect. Sp: Small sample limits ability to rule out no effect.
Ref no.68	Exp	2	1	1	1	P: Uncertain whether adequately powered scale trial could be conducted within acceptable resource constraints. A: Sample likely to be unrepresentative; difficult to translate findings to real-world settings. Se: Small sample and weak outcome measurement limits detection of effect. Sp: Small sample and potential confounding limit ability to rule out no effect.
Ref no.71	Exp	2	2	2	2	P: Adequate power to detect an effect on the intervention package but not to detect effect of individual BCTs. A: Uncertain how well could be applied in practice. Se: Weak outcome measurement relative to what is needed for clinically meaningful health outcomes; difficult to extract individual BCT effectiveness. Sp: Sample too small to rule out small effects of individual BCTs or specific BCT combinations.
Ref no.1	Exp	2	2	1	1	P: Identified a specific BCT but challenging to undertake a study of sufficient size to obtain reliable effect size estimates at sufficiently long follow-up. A: Unclear whether study population is generalizable to populations of interest. Se: Small sample size limits ability to detect BCT effect. Sp: Small sample size limits ability to be confident about no effect.
Ref no.34	Exp	1	1	1	1	P: Setting up a sufficiently well-controlled study with a large enough sample with adequate outcome measures is extremely challenging. A: The setting, methods, and sample make generalizability to the wider population problematic. Se: Weak outcome assessment and comparing two potentially active BCTs against each other limits ability to detect effects. Sp: Use of multiple outcomes and potential for bias in measurement make confident ascription of effects to BCTs problematic.
Ref no.56	Exp	2	2	2	1	P: Study was able to detect intervention effect, but design did not include studying effect of specific BCTs. A: The study methods provide reasonable confidence of generalizability to population of interest but need for consent and self-selection bias may have influenced findings. Se: As an evaluation of an intervention package, identifying contribution of specific BCTs could not be done with confidence, but possible mediating effect of nicotine replacement use provided some indication. Sp: As an evaluation of an intervention package, identifying lack of contribution of specific BCTs was not possible.
Ref no.16	Exp	2	1	1	1	P: Identifying specific BCTs using this methodology would be highly challenging as would securing a sufficient sample size. A: High risk of self-selection bias Se: Small sample size limits ability to detect BCT effects. Sp: Small sample size and potential measurement bias limits ability to rule out ineffective BCTs
Ref no.75	Meta-an	2	2	1	1	P: Limited number of studies available for analysis; large variability on multiple features among the included studies; poor recording of BCTs in intervention and control conditions in studies included. A: Significant risk of self-selection bias in studies included; many studies were more than 10 years old. Se: Able to detect an overall effect of intervention packages but not the effect of specific BCTs. Sp: Unable to rule out effect of specific BCTs
Ref no.76	Meta-reg	3	3	2	2	P: Sufficient studies to draw useful conclusions; somewhat limited by study descriptions. A: Some risk of self-selection bias but otherwise reason to believe findings would be generalizable. Se: Able to detect higher effect sizes associated with inclusion of specific BCTs; not able to detect BCT interactions; not able to rule out confounding by other study features or other BCTs. Sp: Unable to draw confident conclusions about ineffective BCTs.
Ref no.97	Meta-reg	3	3	2	2	P: Sufficient studies to draw useful conclusions; somewhat limited by study descriptions. A: Some risk of self-selection bias but otherwise reason to believe findings would be generalizable. Se: Able to detect higher effect sizes associated with inclusion of specific BCTs; not able to detect BCT interactions; not able to rule out confounding by other study features or other BCTs. Sp: Unable to draw confident conclusions about ineffective BCTs.
Ref no.105	Desc	1	2	1	1	P: Limited number of studies with significant effects on which to draw; large variability in study methods and outcome measures. A: Self-selection bias of studies may limit generalizability; some interventions may not translate to real- world settings. Se: Few effective studies and low quality limits ability to identify effective BCTs. Sp: Few effective studies and low quality limits ability to identify ineffective BCTs.
Ref no.135	Desc	1	2	1	1	P: Most of the included studies were small, lacked a control group, and had a short follow-up. A: Unclear how well BCTs could be applied in current settings as more than 50% of studies were more than 10 years old. Se: Limited ability to detect effective BCTs given the number of studies. Sp: Specific BCTs in effective interventions may not be contributing to the effect.
Ref no.115	Desc	1	2	1	1	P: Able to be conducted within a reasonable time frame and reasonable cost; severely limited by the number and quality of studies available for review. A: Unclear how well BCTs could be applied in current settings as more than 50% of studies were more than 10 years old. Se: Limited ability to detect effective BCTs given the number of studies. Sp: Specific BCTs in effective interventions may not be contributing to the effect.

*Exp* Experimental study; *Obs* Comparative observational study; *Meta-an* Meta-analysis; *Meta-reg* Meta-regression; *Desc* Description of intervention content of effective interventions in RCTs.

*BCTs* behavior change techniques; *P* Practicability; *A* Applicability; *Se* Sensitivity; *Sp* Specificity.

## Discussion

In this scoping review, the most commonly used method was to assess the effectiveness of specific BCTs or BCT combinations in experimental studies. Reviews that characterized the content of effective interventions in terms of their BCTs were also quite common. Analysis of the potential of different methods to identify effective BCTs relevant to specific behaviors and contexts suggested that all had important limitations. This was borne out by applying the criteria of practicability, applicability, sensitivity, and specificity to a sample of the studies in the review.

Since none of the methods adopted appear to be able, in themselves, to provide a high degree of confidence on BCT effectiveness applied to particular behaviors and contexts, the question arises as how to arrive at an *appropriate* level of confidence making use of all the evidence available. A method for doing this should enable a statement of the following kind: “In (an intervention type, including mode of delivery and specific implementation) for (target population and setting) seeking to achieve (behavioral objective), there is (x degree of confidence) that inclusion of (BCT or BCT combination) will increase (a measure of intervention outcome) by (amount) compared with (not including it/including another BCT or BCT combination).” An example would be as follows: “In an interactive website for UK smokers making a quit attempt, there is 95% confidence that rewarding users’ claims of abstinence from smoking with praise will increase 12-month continuous abstinence rates by at least 0.1% compared with not doing so.”

The confidence rating at the heart of these kinds of statements is a subjective confidence arrived at from statistical analyses coupled with judgments based on inference. It is apparent from the findings of this review that judgment will always be required, both for evaluating study quality and for evaluating relevance. Therefore, direct transposition of statistical confidence intervals around effect sizes in studies will never be sufficient. An example of this is that more than 100 high-quality RCTs find that nicotine replacement therapy increases 6-month continuous abstinence rates in smokers making a quit attempts by 60% compared with placebo, with the 95% confidence interval of the meta-analysis ranging from 50% to 70% [39]. However, comparative observational studies find no benefit when smokers use nicotine replacement therapy bought from a pharmacy or general store as opposed to obtaining it from a health professional [40]. Generalization beyond study populations and settings is always required and, therefore, so is judgment.

Given the findings of this review, the question arises as to how to combine evidence most efficiently to arrive at appropriate levels of subjective confidence for the particular behavior, mode, population and setting. As a starting point for this, the following sequence may be considered when assessing the effectiveness of specific BCTs in a given context for a given behavioral outcome:

1 Search for all studies that have used the BCT or BCT combination concerned for the type of behavior of interest, including the full range of methods identified in this review
2. For each study (including reviews),
  (a) record the effect sizes and confidence intervals where these are available
  (b) record the specific outcome measures, features of delivery, target populations and settings.
  (c) record all available evidence on implementation of the BCT/BCT combination, including the specific way it is implemented and any measures of fidelity
  (d) record information that is relevant to a judgment of bias, including conflict of interest statements of authors and study selection or publication bias
3. Starting with the most comprehensively relevant study (e.g., a review where all the features are closest to the specific behavior, mode, population and setting at issue), form a subjective judgment as to the range within which the effect size is likely to lie with what would be considered an acceptable level of subjective confidence (e.g., 95%), taking into account the need to generalize.
4. Then, iteratively update that range with successive studies, weighting each study according to relevance and confidence in its findings.

In essence, this approach follows Bayesian principles of establishing an initial level of confidence in a hypothesis and then updating this incrementally with new information [41]. The extent to which the new information changes the subjective confidence depends on the strength of evidence and its relevance. Strength of evidence will depend on aspects of study design, execution, and reporting. Relevance will depend on how closely the BCT/BCT combination, behavioral outcome, features of delivery, population studied, and setting match those to which one wishes to generalize. Formal methods of arriving at and updating subjective confidence have been used in other areas of policy making [42], and it could be useful to examine how far they could be applied here.

The process being proposed should be considered as ongoing, and it is worth considering how to manage it. The current system of scientific reporting is not well suited to this process since it treats studies in isolation. Even systematic reviews are treated as isolated studies rather than as a process of knowledge accumulation. Scientific papers are currently written as semi-structured narratives relating to a set of research questions. The absence of a coherent, systematic structure linking the studies together creates a dislocation between studies that impedes the efficient accumulation of evidence. Moreover, even with reporting standards such as CONSORT [43] and TIDieR [44], there remain crucial pieces of information that are typically not reported or are reported in a way that does not permit the information to be used in the knowledge accumulation process. The upcoming CONSORT extension on psychological and social interventions [45] will mitigate this problem, but there will still be important gaps. One important piece of information is the extent to which BCTs are delivered as planned, in terms of both extent and quality. We know from studies investigating behavioral support for smoking cessation and physical activity that fidelity of delivery of BCTs can be poor with often fewer than 50% of BCTs in intervention protocols delivered in practice [46–49]. This is compounded by the problem of selective reporting of intervention protocols in published reports with an analysis of studies synthesized in Cochrane reviews of behavioral support for smoking cessation finding that fewer than 50% of protocol-specified BCTs were reported in the published article [50]. Thus, one can see that there is a possibility that a different set of BCTs may be delivered in practice than are reported in the published article, with devastating consequences for the reliability of evidence syntheses (for a broader discussion of these issues, please see a discussion in Health Psychology Review, e.g [51–53].).

In terms of quality of BCTs delivered, BCTs may be delivered wholly or in part. This has been acknowledged in the coding scheme developed by Michie et al. in which BCTs were coded ‘++’ if the BCT was judged to be present beyond all reasonable doubt, with clear evidence available and ‘+’ if they were present in all probability but the evidence was not clear [9]. This distinction has been observed both in coding written materials (e.g., intervention protocols and manuals and published reports) and in recorded intervention sessions (e.g., audiotape recordings). An example of the latter is the quality of delivering “goal setting” in behavioral support for smoking cessation [54]. Analysis of session transcripts showed a large variation in how advisers enacted the protocol concerning setting a quit date, from which a reliable 10-item scale of the quality of delivery of this BCT was developed. Applying this to 85 transcribed behavioral support sessions found that higher quality goal setting increased reported quit attempts (*p* < .001; OR = 2.60, 95% CI: 1.54 – 4.40) and that the scale components “set a clear quit date” (χ^2^ (2, *N* = 85) = 22.3, *p* < .001) and “within an appropriate timeframe” (χ^2^ (2, N = 85) = 15.5, *p* < .001) were independently associated with quit attempts. Although this method has been applied for only one of the 93 BCTs, it demonstrates the utility of pursuing this line of research.

To more fully understand the association between the effects of BCTs on behavior, it is necessary to codify knowledge about other aspects of behavior change interventions and include this in analyses of BCT effectiveness. One approach to the process of accumulating knowledge in this area is to construct an “ontology.” Ontologies are sets of elements or constructs and relationships between them which codify our collective knowledge, reflect consensus on concepts, terms, relationships, and specify and formalize them. Such relationships can be anything from semantic to causal. In the case of making judgments about effectiveness of BCTs/BCT combinations, each new piece of information could update an ontology expressing confidence about effect size for specified behavioral targets when implemented in specified ways, using particular modes of delivery to given target populations in defined settings. Such a “behavior change ontology” could also link BCTs to a set of mechanisms of action and a set of theories [55].

We have begun the work of extending the BCT taxonomy to build a more elaborated “Behavior Change Intervention Ontology.” This is a conceptual structure for systematically representing, sorting and linking the “elements” of behavior change interventions, that is, the content, features of delivery, target behavior, setting, and mechanism of action with effect sizes. It brings these pieces together into a framework that can encourage commonality of conceptualization and terminology across the scientific community and guide the generation, access, and application of evidence to answer the question “What works, how well, for whom, in what settings, for what behaviors, and why?”

**Fig. 2:**
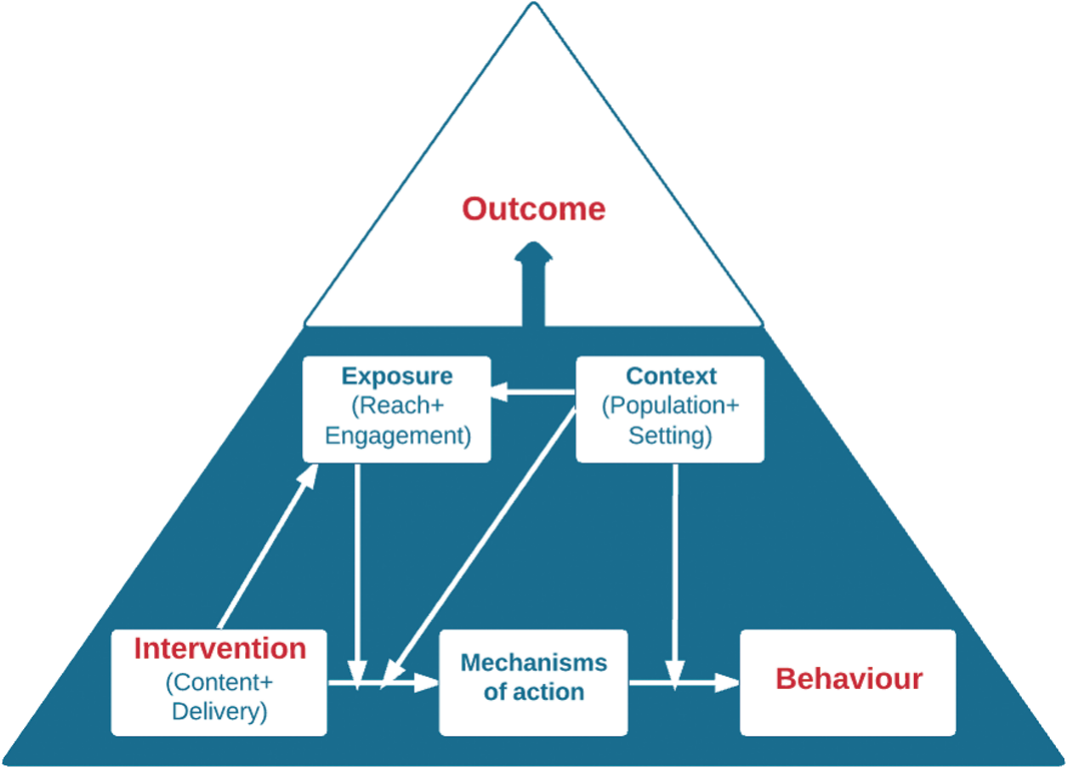
Ontology of behavior change interventions[64]

Note: The diagram represents how features of an intervention (its content and the way that the content is delivered) interact with context (the features of the target population and the setting) and features of the target behavior and its measurement to produce a particular effect size. The above features are moderated and mediated by exposure and mechanisms of action, respectively.

We show the top level of such an ontology, specifying key elements and their relationships in Fig. 2 [56]. It should be noted that this ontology has been developed as a method for organizing evidence about behavior change interventions rather than as a representation of behavior in context in real time. For this purpose, additional parameters and feedback loops would need to be added to represent the temporal dimension. The Human Behavior Change project (www.humanbehaviourchange.org), a 4-year collaboration between behavioral, computer, and information scientists, is building the Behavior Change Intervention Ontology building on current work by Michie et al. [57]. Intervention content has been specified in terms of 93 BCTs within 16 groupings [3, 9]. A taxonomy of modes of delivery has been developed with 39 items at 4 levels; it is currently undergoing expert validation. A simple classification of mechanisms of action drawn from theory has been developed in a multidisciplinary consensus exercise to give an integrative framework of 14 domains of theoretical constructs, the Theoretical Domains Framework [58, 59]. A much more ambitious project is currently underway to organize the >1,700 theoretical constructs from 83 theories identified in a multidisciplinary literature review of behavior change theories [60, 61]. These theories had a mean of 21 constructs, ranging from 5 to 84, with many being the same as or similar to those in other theories. The task of defining these constructs, and the relationships between them, within and across theories, is in progress. The current structure of types of mechanisms of action across theories has 3 levels of hierarchy within 14 domains [56]. Within theories, we have identified 14 possible relationships between constructs; these have been converted into diagrammatic specification, which is machine readable, and we are working with computer scientists to investigate extracting from these data one or more “canonical” theories to encapsulate the overlaps across the 83 theories.

The links between BCTs and frequently identified mechanisms of action are being investigated in a cross-disciplinary, international project funded by the UK’s Medical Research Council, described in a published protocol paper [62]. We are collaborating in a project, led by Kai Larsen, to develop a taxonomy of behaviors, which takes as a starting point the World Health Organization’s International Classification of Functioning [63].This is a mammoth undertaking, requiring expertise from both behavioral and computer sciences to build such an ontology to make sense of the vast and rapidly accelerating volume of published relevant literature. However, like an encyclopedia, it would have the considerable benefit of becoming useful almost immediately and gradually increasing in value as it grew. By linking with machine learning, it has the potential to efficiently and effectively harness evidence in real time, support the rapid testing and refinement of theories, and make evidence useable and useful to researchers, practitioners, and policymakers. The Human Behavior Change Project will develop shared concepts, terms, and relationships between those concepts to precisely specify not just the content of behavioral interventions (BCTs) but all the mediators and moderators that will allow us to understand their effects on behaviors, specified at different levels of granularity. In this way, it will revolutionize our ability to synthesize evidence about behavior change in real time and to generate new insights about behavior change. It will include a searchable, up-to-date database of evidence that will allow people to design and implement the best possible behavioral intervention for their circumstances.

To take us back to the focus of this paper, we can conclude that research evaluating the effectiveness of BCTs/BCT combinations uses a range of experimental and observational methods, each with strengths and limitations. Making judgments of the effectiveness of a BCT/BCT combination for a given behavior, delivered in a particular way, to a given target population in a given setting requires synthesis of information from diverse sources to arrive at a subjective confidence estimate. A process for achieving this is proposed together with a paradigm shift in the way research in this area is conducted, reported, and synthesized. This is ambitious, but given the importance of behavior change to the welfare of the world’s population, it is worth putting considerable resources into achieving it.

## Supplementary Material

Supplementary material is available at *Translational Behavioral Medicine* online.

Supplementary File
