# Supplementary material for: Evaluating the effectiveness of behavior change techniques in health-related behavior: a scoping review of methods used
Source: Transl Behav Med. 2018 Feb 10;8(2):212–24. doi: 10.1093/tbm/ibx019 (PMC6062857; doi:10.1093/tbm/ibx019)
Supplement: Supplementary File [file ibx019_suppl_supplementary_file.docx]

Supplementary File: References to included studies, assigned to each category of effectiveness evaluation methods

**Experiments (including RCTs)**

1. Bélanger-Gravel, A., Godin, G., Bilodeau, A., & Poirier, P. (2013). The effect of implementation intentions on physical activity among obese older adults: A randomised control study. *Psychology & Health, 28*(2), 217-233.
2. Booth, A. R., Norman, P., Goyder, E., Harris, P. R., & Campbell, M. J. (2014). Pilot study of a brief intervention based on the theory of planned behaviour and self-identity to increase chlamydia testing among young people living in deprived areas. British *Journal of Health Psychology, 19*(3), 636-651.
3. Brown, J., Michie, S., Geraghty, A. W., Miller, S., Yardley, L., Gardner, B., . . . West, R. (2012). A pilot study of StopAdvisor: A theory-based interactive internet-based smoking cessation intervention aimed across the social spectrum. *Addictive Behaviors, 37*(12), 1365-1370. doi: 10.1016/j.addbeh.2012.05.016
4. Bukowska-Durawa, A., Haynes, C., & Luszczynska, A. (2010). Plans not needed if you have high and stable self-efficacy: Planning intervention and snack intake in the context of self-efficacy trajectories. *Polish Psychological Bulletin, 41*(3), 91-97.
5. Cheval, B., Sarrazin, P., Isoard-Gautheur, S., Radel, R., & Friese, M. (2015). Reflective and impulsive processes explain (in)effectiveness of messages promoting physical activity: A randomized controlled trial*. Health Psychology, 34*(1), 10-19. doi: 10.1037/hea0000102
6. Contzen, N., Meili, I. H., & Mosler, H.-J. (2015). Changing handwashing behaviour in southern Ethiopia: A longitudinal study on infrastructural and commitment interventions. *Social Science & Medicine, 124*, 103-114. doi: 10.1016/j.socscimed.2014.11.006
7. Cullen, K. W., Thompson, D., Boushey, C., Konzelmann, K., & Chen, T.-A. (2013). Evaluation of a web-based program promoting healthy eating and physical activity for adolescents: Teen Choice: Food and Fitness. *Health Education Research, 28*(4), 704-714.doi:10.1093/her/cyt059
8. De Greef, K., Deforche, B., Tudor-Locke, C., & De Bourdeaudhuij, I. (2010). A cognitive-behavioural pedometer-based group intervention on physical activity and sedentary behaviour in individuals with type 2 diabetes. *Health Education Research, 25*(5), 724-736. doi: 10.1093/her/cyq017
9. De Janssen, V., Gucht, V., van Exel, H., & Maes, S. (2014). A self-regulation lifestyle program for post-cardiac rehabilitation patients has long-term effects on exercise adherence. *Journal of Behavioral Medicine, 37*(2), 308-321. doi:10.1007/s10865-012-9489-y
10. Devi, R., Powell, J., & Singh, S. (2014). A web-based program improves physical activity outcomes in a primary care angina population: Randomized controlled trial. *Journal of Medical Internet Research, 16*(9), 37-49. doi: 10.2196/jmir.3340
11. Dickie, R., & Finegan, S. (1977). The long-term effects of self-recording on academic behavior rate in an emotionally disturbed boy. *SALT: School Applications of Learning Theory, 9*(4), 38-48.
12. Ernsting, A., Schwarzer, R., Lippke, S., & Schneider, M. (2013). 'I do not need a flu shot because I lead a healthy lifestyle': Compensatory health beliefs make vaccination less likely. *Journal of Health Psychology, 18*(6), 825-836. doi:10.1177/1359105312455076
13. Fletcher, A., Wolfenden, L., Wyse, R., Bowman, J., McElduff, P., & Duncan, S. (2013). A randomised controlled trial and mediation analysis of the 'Healthy Habits', telephone-based dietary intervention for preschool children. *International Journal of Behavioral Nutrition and Physical Activity, 10*. doi: 10.1186/1479-5868-10-43
14. Free, C., Knight, R., Robertson, S., Whittaker, R., Edwards, P., Zhou, W., . . . Roberts, I. (2011). Smoking cessation support delivered via mobile phone text messaging (txt2stop): A single-blind, randomised trial. *The Lancet, 378*(9785), 49-55. doi:10.1016/S0140-6736(11)60701-0
15. Gaston, A., & Prapavessis, H. (2014). Using a combined protection motivation theory and health action process approach intervention to promote exercise during pregnancy. *Journal of Behavioral Medicine, 37*(2), 173-184. doi:10.1007/s10865-012-9477-2
16. George, S. M. S., Wilson, D. K., Schneider, E. M., & Alia, K. A. (2013). Project SHINE: Effects of parent-adolescent communication on sedentary behavior in African American adolescents. *Journal of Pediatric Psychology, 38*(9), 997-1009.
17. Gholami, M., Lange, D., Luszczynska, A., Knoll, N., & Schwarzer, R. (2013). A dietary planning intervention increases fruit consumption in Iranian women. *Appetite, 63,* 1-6. doi:10.1016/j.appet.2012.12.005
18. Gholami, M., Wiedemann, A., Knoll, N., & Schwarzer, R. (2015). Mothers improve their daughters' vegetable intake: A randomized controlled trial. *Psychology, Health & Medicine, 20*(1), 1-7. doi:10.1080/13548506.2014.902485
19. Guillaumie, L., Godin, G., Manderscheid, J.-C., Spitz, E., & Muller, L. (2012). The impact of self-efficacy and implementation intentions-based interventions on fruit and vegetable intake among adults. *Psychology & Health, 27*(1), 30-50. doi:10.1080/08870446.2010.541910
20. Hagger, M. S., Lonsdale, A., & Chatzisarantis, N. L. D. (2012). A theory-based intervention to reduce alcohol drinking in excess of guideline limits among undergraduate students. *British Journal of Health Psychology, 17*, 18-43. doi: 10.1111/j.2044-8287.2010.02011.x
21. Hagger, M. S., Lonsdale, A., Koka, A., Hein, V., Pasi, H., Lintunen, T., & Chatzisarantis, N. L. (2012). An intervention to reduce alcohol consumption in undergraduate students using implementation intentions and mental simulations: A cross-national study. *International Journal of Behavioral Medicine, 19*(1), 82-96. doi:10.1007/s12529-011-9163-8
22. Harris, K. B., & Miller, W. R. (1990). Behavioral self-control training for problem drinkers: Components of efficacy. *Psychology of Addictive Behaviors, 4*(2), 82-90.
23. Hill, C., Abraham, C., & Wright, D. B. (2007). Can theory-based messages in combination with cognitive prompts promote exercise in classroom settings? *Social Science & Medicine, 65*(5), 1049-1058. doi: 10.1016/j.socscimed.2007.04.024
24. Huber, A. C., & Mosler, H.-J. (2013). Determining the differential preferences of users of two fluoride-free water options in rural Ethiopia. *Journal of Public Health, 21*(2), 183-192.
25. Inauen, J., & Mosler, H.-J. (2014). Developing and testing theory-based and evidence-based interventions to promote switching to arsenic-safe wells in Bangladesh. *Journal of Health Psychology, 19*(12), 1483-1498.
26. Ipsen, C., Ruggiero, C., Rigles, B., Campbell, D., & Arnold, N. (2014). Evaluation of an online health promotion program for vocational rehabilitation consumers. *Rehabilitation Psychology, 59*(2), 125-135.
27. Irvine, A., Gelatt, V. A., Seeley, J. R., Macfarlane, P., & Gau, J. M. (2013). Web-based intervention to promote physical activity by sedentary older adults: Randomized controlled trial. *Journal of Medical Internet Research, 15*(2), 18-33.
28. Joost, R., Doerje, F., Schwitulla, J., Eckardt, K.-U., & Hugo, C. (2014). Intensified pharmaceutical care is improving immunosuppressive medication adherence in kidney transplant recipients during the first post-transplant year: a quasi-experimental study. *Nephrology Dialysis Transplantation, 29*(8), 1597-1607. doi: 10.1093/ndt/gfu207
29. Kelders, S. M., Van Gemert-Pijnen, J. E., Werkman, A., Nijland, N., & Seydel, E. R. (2011). Effectiveness of a web-based intervention aimed at healthy dietary and physical activity behavior: A randomized controlled trial about users and usage. *Journal of Medical Internet Research, 13*(2), 3-18.
30. Kiernan, M., Brown, S. D., Schoffman, D. E., Lee, K., King, A. C., Taylor, C., . . . Perri, M. G. (2013). Promoting healthy weight with "stability skills first": A randomized trial. *Journal of Consulting and Clinical Psychology, 81*(2), 336-346.
31. Koring, M., Richert, J., Parschau, L., Ernsting, A., Lippke, S., & Schwarzer, R. (2012). A combined planning and self-efficacy intervention to promote physical activity: A multiple mediation analysis. *Psychology, Health & Medicine, 17*(4), 488-498.
32. Kothe, E., Mullan, B., & Butow, P. (2012). Promoting fruit and vegetable consumption. Testing an intervention based on the theory of planned behaviour. *Appetite, 58*(3), 997-1004.
33. Lange, D., Richert, J., Koring, M., Knoll, N., Schwarzer, R., & Lippke, S. (2013). Self-regulation prompts can increase fruit consumption: A one-hour randomised controlled online trial. *Psychology & Health, 28*(5), 533-545.
34. Latif, H., Watson, K., Nguyen, N., Thompson, D., Baranowski, J., Jago, R., . . . Baranowski, T. (2011). Effects of goal setting on dietary and physical activity changes in the Boy Scout Badge projects. *Health Education & Behavior, 38*(5), 521-529.
35. Lemmens, K. P., Ruiter, R. A., Abraham, C., Veldhuizen, I. J., & Schaalma, H. P. (2010). Motivating blood donors to recruit new donors: Experimental evaluation of an evidence-based behavior change intervention. *Health Psychology, 29*(6), 601-609.
36. Lewis, B. A., Williams, D. M., Martinson, B. C., Dunsiger, S., & Marcus, B. H. (2013). Healthy for life: A randomized trial examining physical activity outcomes and psychosocial mediators. *Annals of Behavioral Medicine, 45*(2), 203-212.
37. Lubans, D. R., Plotnikoff, R. C., Jung, M., Eves, N., & Sigal, R. (2012). Testing mediator variables in a resistance training intervention for obese adults with type 2 diabetes. *Psychology & Health, 27*(12), 1388-1404.
38. Luszczynska, A., Schwarzer, R., Lippke, S., & Mazurkiewicz, M. (2011). Self-efficacy as a moderator of the planning-behaviour relationship in interventions designed to promote physical activity. *Psychology & Health, 26*(2), 151-166.
39. Madigan, C. D., Jolly, K., Lewis, A. L., Aveyard, P., & Daley, A. J. (2014). A randomised controlled trial of the effectiveness of self-weighing as a weight loss intervention. *The International Journal of Behavioral Nutrition and Physical Activity, 11*, 125.
40. Mailey, E. L., & McAuley, E. (2014). Impact of a brief intervention on physical activity and social cognitive determinants among working mothers: A randomized trial. *Journal of Behavioral Medicine, 37*(2), 343-355.
41. McEachan, R. R., Lawton, R. J., Jackson, C., Conner, M., Meads, D. M., & West, R. M. (2011). Testing a workplace physical activity intervention: A cluster randomized controlled trial. *The International Journal of Behavioral Nutrition and Physical Activity, 8*, 29.
42. McGowan, E. L., Prapavessis, H., Campbell, N., Gray, C., & Elkayam, J. (2012). The effect of a multifaceted efficacy intervention on exercise behavior in relatives of colon cancer patients*. International Journal of Behavioral Medicine, 19*(4), 550-562.
43. McMurdo, M. E., Sugden, J., Argo, I., Boyle, P., Johnston, D. W., Sniehotta, F. F., & Donnan, P. T. (2010). Do pedometers increase physical activity in sedentary older women? A randomized controlled trial. *Journal of the American Geriatrics Society, 58*(11), 2099-2106.
44. Meng, K., Seekatz, B., Haug, G., Mosler, G., Schwaab, B., Worringen, U., & Faller, H. (2014). Evaluation of a standardized patient education program for inpatient cardiac rehabilitation: Impact on illness knowledge and self-management behaviors up to 1 year. *Health Education Research, 29*(2), 235-246.
45. Morgan, P. J., Collins, C. E., Plotnikoff, R. C., Cook, A. T., Berthon, B., Mitchell, S., & Callister, R. (2011). Efficacy of a workplace-based weight loss program for overweight male shift workers: The Workplace POWER (Preventing Obesity Without Eating like a Rabbit) randomized controlled trial. *Preventive Medicine: An International Journal Devoted to Practice and Theory, 52*(5), 317-325.
46. Morgan, P., Lubans, D., Callister, R., Okely, A., Burrows, T., Fletcher, R., & Collins, C. (2011). The 'Healthy Dads, Healthy Kids' randomized controlled trial: Efficacy of a healthy lifestyle program for overweight fathers and their children*. International Journal of Obesity, 35*(3), 436-447.
47. Morrison, R., Reilly, J. J., Penpraze, V., Westgarth, C., Ward, D. S., Mutrie, N., . . . Yam, P. S. (2013). Children, parents and pets exercising together (CPET): exploratory randomised controlled trial. *BMC Public Health, 13,* 1096. doi: 10.1186/1471-2458-13-1096
48. Newbury-Birch, D., Coulton, S., Bland, M., Cassidy, P., Dale, V., Deluca, P., . . . Drummond, C. (2014). Alcohol screening and brief interventions for offenders in the probation setting (SIPS Trial): A pragmatic multicentre cluster randomized controlled trial. *Alcohol and Alcoholism, 49*(5), 540-548.
49. O'Carroll, R. E., Chambers, J. A., Dennis, M., Sudlow, C., & Johnston, M. (2014). Improving medication adherence in stroke survivors: Mediators and moderators of treatment effects. *Health Psychology, 33*(10), 1241-1250.
50. Payaprom, Y., Bennett, P., Alabaster, E., & Tantipong, H. (2011). Using the Health Action Process Approach and implementation intentions to increase flu vaccine uptake in high risk Thai individuals: A controlled before-after trial. *Health Psychology, 30*(4), 492-500.
51. Plotnikoff, R. C., Lubans, D. R., Penfold, C. M., & Courneya, K. S. (2014). Testing mediator variables in a physical activity intervention for women with type 2 diabetes. *Psychology of Sport and Exercise, 15*(1), 1-8.
52. Pomp, S., Fleig, L., Schwarzer, R., & Lippke, S. (2013). Effects of a self-regulation intervention on exercise are moderated by depressive symptoms: A quasi-experimental study. *International Journal of Clinical and Health Psychology, 13*(1), 1-8.
53. Rackow, P., Scholz, U., & Hornung, R. (2014). Effects of a new sports companion on received social support and physical exercise: An intervention study. *Applied Psychology: Health and Well-Being, 6*(3), 300-317.
54. Robroek, S. J., Polinder, S., Bredt, F. J., & Burdorf, A. (2012). Cost-effectiveness of a long-term Internet-delivered worksite health promotion programme on physical activity and nutrition: A cluster randomized controlled trial. *Health Education Research, 27*(3), 399-410.
55. Santer, M., Muller, I., Yardley, L., Burgess, H., Selinger, H., Stuart, B. L., & Little, P. (2014). Supporting self-care for families of children with eczema with a Web-based intervention plus health care professional support: Pilot randomized controlled trial. *Journal of Medical Internet Research, 16*(3), 53-67.
56. Schuck, K., Bricker, J. B., Otten, R., Kleinjan, M., Brandon, T. H., & Engels, R. C. (2014). Effectiveness of proactive quitline counselling for smoking parents recruited through primary schools: Results of a randomized controlled trial. *Addiction, 109*(5), 830-841.
57. Schulz, D. N., Candel, M. J., Kremers, S. P., Reinwand, D. A., Jander, A., & de Vries, H. (2013). Effects of a Web-based tailored intervention to reduce alcohol consumption in adults: Randomized controlled trial. *Journal of Medical Internet Research, 15*(9), 53-68.
58. Schulz, D. N., Kremers, S. P., Vandelanotte, C., van Adrichem, M. J., Schneide, F., Candel, M. J., & de Vries, H. (2014). Effects of a Web-based tailored multiple-lifestyle intervention for adults: A two-year randomized controlled trial comparing sequential and simultaneous delivery modes. *Journal of Medical Internet Research, 16*(1), 48-65.
59. Schweier, R., Romppel, M., Richter, C., Hoberg, E., Hahmann, H., Scherwinski, I., . . . Grande, G. (2014). A Web-based peer-modeling intervention aimed at lifestyle changes in patients with coronary heart disease and chronic back pain: Sequential controlled trial. *Journal of Medical Internet Research, 16*(7), 29-42.
60. Scott, S. E., Khwaja, M., Low, E. L., Weinman, J., & Grunfeld, E. A. (2012). A randomised controlled trial of a pilot intervention to encourage early presentation of oral cancer in high risk groups. *Patient Education and Counseling, 88*(2), 241-248.
61. Seghers, J., Van Hoecke, A.-S., Schotte, A., Opdenacker, J., & Boen, F. (2014). The added value of a brief self-efficacy coaching on the effectiveness of a 12-week physical activity program. *Journal of Physical Activity & Health, 11*(1), 18-29.
62. Sniehotta, F. F., Presseau, J., Hobbs, N., & Araujo-Soares, V. (2012). Testing self-regulation interventions to increase walking using factorial randomized N-of-1 trials. *Health Psychology, 31*(6), 733-737. doi: 10.1037/a0027337
63. Suresh, R., Jones, K. C., Timothy, J., & Asimakopoulou, K. (2012). An exploratory study into whether self-monitoring improves adherence to daily flossing among dental patients. *Journal of Public Health Dentistry, 72*(1), 1-7. doi: 10.1111/j.1752-7325.2011.00274.x
64. Tapper, K., Jiga-Boy, G., Maio, G. R., Haddock, G., & Lewis, M. (2014). Development and preliminary evaluation of an Internet-based healthy eating program: Randomized controlled trial. *Journal of Medical Internet Research, 16*(10), 12-27.
65. Taylor, A. H., Thompson, T. P., Greaves, C. J., Taylor, R. S., Green, C., Warren, F. C., . . . West, R. (2014). A pilot randomised trial to assess the methods and procedures for evaluating the clinical effectiveness and cost-effectiveness of Exercise Assisted Reduction then Stop (EARS) among disadvantaged smokers. *Health Technology Assessment, 18*(4), 1-+. doi: 10.3310/hta18040
66. van Genugten, L., van Empelen, P., Boon, B., Borsboom, G., Visscher, T., & Oenema, A. (2012). Results from an online computer-tailored weight management intervention for overweight adults: Randomized controlled trial. *Journal of Medical Internet Research, 14*(2), 100-114.
67. Vernon, S. W., Bartholomew, L. K., McQueen, A., Bettencourt, J. L., Greisinger, A., Coan, S. P., . . . Myers, R. (2011). A randomized controlled trial of a tailored interactive computer-delivered intervention to promote colorectal cancer screening: Sometimes more is just the same. *Annals of Behavioral Medicine, 41*(3), 284-299.
68. Verwey, R., van der Weegen, S., Spreeuwenberg, M., Tange, H., van der Weijden, T., & de Witte, L. (2014). A pilot study of a tool to stimulate physical activity in patients with COPD or type 2 diabetes in primary care. *Journal of Telemedicine and Telecare, 20*(1), 29-34. doi: 10.1177/1357633x13519057
69. Watson, A., Bickmore, T., Cange, A., Kulshreshtha, A., & Kvedar, J. (2012). An Internet-based virtual coach to promote physical activity adherence in overweight adults: Randomized controlled trial. *Journal of Medical Internet Research, 14*(1), 44-55.
70. Whittaker, R., Dorey, E., Bramley, D., Bullen, C., Denny, S., Elley, C. R., . . . Salmon, P. (2011). A theory-based video messaging mobile phone intervention for smoking cessation: randomized controlled trial. *Journal of Medical Internet Research, 13*(1), e10. doi: 10.2196/jmir.1553
71. Wolfenden, L., Wyse, R., Campbell, E., Brennan, L., Campbell, K. J., Fletcher, A., . . . Heard, T. R. (2014). Randomized controlled trial of a telephone-based intervention for child fruit and vegetable intake: long-term follow-up. *American Journal of Clinical Nutrition, 99*(3), 543-550. doi: 10.3945/ajcn.113.071738
72. Wolff, J. K., Warner, L. M., Ziegelmann, J. P., & Wurm, S. (2014). What do targeting positive views on ageing add to a physical activity intervention in older adults? Results from a randomised controlled trial. *Psychology & Health, 29*(8), 915-932.
73. Yardley, L., Miller, S., Schlotz, W., & Little, P. (2011). Evaluation of a web-based intervention to promote hand hygiene: Exploratory randomized controlled trial. *Journal of Medical Internet Research, 13*(4), 218-231.

**Meta-analyses of experimental studies**

1. Arnott, B., Rehackova, L., Errington, L., Sniehotta, F. F., Roberts, J., & Araujo-Soares, V. (2014). Efficacy of behavioural interventions for transport behaviour change: Systematic review, meta-analysis and intervention coding. *The International Journal of Behavioral Nutrition and Physical Activity, 11*, 133.
2. Avery, L., Flynn, D., van Wersch, A., Sniehotta, F. F., & Trenell, M. I. (2012). Changing physical activity behavior in type 2 diabetes: a systematic review and meta-analysis of behavioral interventions. *Diabetes Care, 35*(12), 2681-2689. doi: 10.2337/dc11-2452
3. Bartlett, Y. K., Sheeran, P., & Hawley, M. S. (2014). Effective behaviour change techniques in smoking cessation interventions for people with chronic obstructive pulmonary disease: a meta-analysis. *British Journal of Health Psychology, 19*(1), 181-203. doi: 10.1111/bjhp.12071
4. Bélanger-Gravel, A., Godin, G., & Amireault, S. (2013). A meta-analytic review of the effect of implementation intentions on physical activity*. Health Psychology Review, 7*(1), 23-54.
5. Bishop, F. L., Fenge-Davies, A. L., Kirby, S., & Geraghty, A. W. A. (2015). Context effects and behaviour change techniques in randomised trials: A systematic review using the example of trials to increase adherence to physical activity in musculoskeletal pain. *Psychology & Health, 30*(1), 104-121. doi: 10.1080/08870446.2014.953529
6. Bull, E., Dombrowski, S. U., & Johnston, M. (2013). Behaviour change techniques within diet, activity and smoking interventions for low-income groups: A systematic review. *Psychology Health, 28*, 75-75.
7. French, D. P., Olander, E. K., Chisholm, A., & Mc Sharry, J. (2014). Which Behaviour Change Techniques Are Most Effective at Increasing Older Adults' Self-Efficacy and Physical Activity Behaviour? A Systematic Review. *Annals of Behavioral Medicine.* doi: 10.1007/s12160-014-9593-z
8. Hill, B., Skouteris, H., & Fuller-Tyszkiewicz, M. (2013). Interventions designed to limit gestational weight gain: a systematic review of theory and meta-analysis of intervention components. *Obesity Review, 14*(6), 435-450. doi: 10.1111/obr.12022
9. Johnson, B. T., Lennon, C. A., Huedo-Medina, T. B., Spina, M., Sagherian, M., & Ballester, E. (2013). Behaviour change techniques succeed best under optimal circumstances. *Psychology & Health, 28*, 15-15.
10. Knittle, K., Maes, S., & de Gucht, V. (2010). Psychological interventions for rheumatoid arthritis: examining the role of self-regulation with a systematic review and meta-analysis of randomized controlled trials. *Arthritis Care Research (Hoboken)*, 62(10), 1460-1472. doi: 10.1002/acr.20251
11. Lara, J., Evans, E. H., O'Brien, N., Moynihan, P. J., Meyer, T. D., Adamson, A. J., . . . Mathers, J. C. (2014). Association of behaviour change techniques with effectiveness of dietary interventions among adults of retirement age: a systematic review and meta-analysis of randomised controlled trials. *Bmc Medicine, 12*. doi: 10.1186/s12916-014-0177-3
12. Meader, N., Semaan, S., Halton, M., Bhatti, H., Chan, M., Llewellyn, A., & Des Jarlais, D. C. (2013). An international systematic review and meta-analysis of multisession psychosocial interventions compared with educational or minimal interventions on the HIV sex risk behaviors of people who use drugs. *Aids and Behavior, 17*(6), 1963-1978.
13. Olander, E. K., Fletcher, H., Williams, S., Atkinson, L., Turner, A., & French, D. P. (2013). What are the most effective techniques in changing obese individuals' physical activity self-efficacy and behaviour: a systematic review and meta-analysis. *International Journal of Behavioral Nutrition and Physical Activity, 10,* 29. doi: 10.1186/1479-5868-10-29
14. Peters, G.-J. Y., Ruiter, R. A., & Kok, G. (2013). Threatening communication: A critical re-analysis and a revised meta-analytic test of fear appeal theory. *Health Psychology Review, 7*(Suppl 1), S8-S31.
15. Webb, T. L., Joseph, J., Yardley, L., & Michie, S. (2010). Using the internet to promote health behavior change: a systematic review and meta-analysis of the impact of theoretical basis, use of behavior change techniques, and mode of delivery on efficacy. *Journal of Medical Internet Research, 12*(1), e4. doi: 10.2196/jmir.1376
16. Williams, S., & French, D. (2011). What are the most effective intervention techniques for changing physical activity self-efficacy and physical activity behavior-and are they the same? *Health Education Research, 26*(2), 308-322.

**Correlational studies**

1. Hankonen, N., Sutton, S., Prevost, A. T., Simmons, R. K., Griffin, S. J., Kinmonth, A. L., & Hardeman, W. (2014). Which Behavior Change Techniques are Associated with Changes in Physical Activity, Diet and Body Mass Index in People with Recently Diagnosed Diabetes? *Annals of Behavioral Medicine*. doi: 10.1007/s12160-014-9624-9
2. Murray, R. L., Szatkowski, L., & Ussher, M. (2013). Evaluation of a refined, nationally disseminated self-help intervention for smoking cessation ("quit kit-2"). *Nicotine and Tobacco Research*, 15(8), 1365-1371. doi: 10.1093/ntr/nts286
3. West, R., Evans, A., & Michie, S. (2011). Behavior change techniques used in group-based behavioral support by the English stop-smoking services and preliminary assessment of association with short-term quit outcomes. *Nicotine and Tobacco Research*, 13(12), 1316-1320. doi: 10.1093/ntr/ntr120
4. West, R., Walia, A., Hyder, N., Shahab, L., & Michie, S. (2010). Behavior change techniques used by the English Stop Smoking Services and their associations with short-term quit outcomes. *Nicotine and Tobacco Research*, 12(7), 742-747. doi: 10.1093/ntr/ntq074

**Meta-regressions**

1. Abraham, C., Good, A., Huedo-Medina, T. B., Warren, M. R. & Johnson, B. T. (2012). Reliability and Utility of the SHARP Taxonomy of Behaviour Change Techniques. *Psychology and Health*, 27, 1-2.
2. Denford, S., Taylor, R. S., Campbell, J. L., & Greaves, C. J. (2013). Effective Behavior Change Techniques in Asthma Self-Care Interventions: Systematic Review and Meta-Regression. *Health Psychology*, 33(7), 577-587. doi: 10.1037/a0033080
3. Dombrowski, S. U., Sniehotta, F. F., Avenell, A., Johnston, M., MacLennan, G., & Araujo-Soares, V. (2012). Identifying active ingredients in complex behavioural interventions for obese adults with obesity-related co-morbidities or additional risk factors for co-morbidities: a systematic review. *Health Psychology Review*, 6(1), 7-32. doi: 10.1080/17437199.2010.513298
4. Hartmann-Boyce, J., Johns, D. J., Jebb, S. A., & Aveyard, P. (2014). Effect of behavioural techniques and delivery mode on effectiveness of weight management: systematic review, meta-analysis and meta-regression. *Obesity Reviews*. doi: 10.1111/obr.12165
5. Lara, J., Evans, E., Hobbs, N., Meyer, T. D., Sniehotta, F. F., & Mathers, J. C. (2013). The effect of behaviour change techniques on interventions promoting components of a Mediterranean diet among adults of retirement age. *Proceedings of the Nutrition Society*, 72(OCE4), E307-E307. doi: 10.1017/s0029665113003340
6. Michie, S., Abraham, C., Whittington, C., McAteer, J., & Gupta, S. (2009). Effective Techniques in Healthy Eating and Physical Activity Interventions: A Meta-Regression. *Health Psychology*, 28(6), 690-701. doi: 10.1037/a0016136
7. Michie, S., Whittington, C., Hamoudi, Z., Zarnani, F., Tober, G., & West, R. (2012). Identification of behaviour change techniques to reduce excessive alcohol consumption. *Addiction*, 107(8), 1431-1440. doi: 10.1111/j.1360-0443.2012.03845.x
8. Stavri, Z., & Beard, E. (2009). Applying a taxonomy of behaviour change techniques to smoking cessation: Are published descriptions adequate? *Psychology and Health*, 24, 375-375.
9. Taylor, N., Conner, M., & Lawton, R. (2012). The impact of theory on the effectiveness of worksite physical activity interventions: a meta-analysis and meta-regression. *Health Psychology Review, 6*(1), 33-73. doi: 10.1080/17437199.2010.533441

**Meta-CART (Classification and Regression Trees)**

1. Dusseldorp, E., van Genugten, L., van Buuren, S., Verheijden, M. W., & van Empelen, P. (2013). Combinations of Techniques That Effectively Change Health Behavior: Evidence From Meta-CART Analysis. *Health Psychology*. Advance online publication. doi: 10.1037/hea0000018

**Characterising effective interventions**

1. Angela, R., Vera, A.-S., & Falko, S. (2011). Interventions promoting sun-protective behaviours: An analysis of effective behaviour change techniques and modes of delivery. *Psychology & Health*, 26, 55-55.
2. Bélanger-Gravel, A., Godin, G., Vezina-Im, L. A., Amireault, S., & Poirier, P. (2011). The effect of theory-based interventions on physical activity participation among overweight/obese individuals: a systematic review. Obesity Reviews, 12(6), 430-439. doi: 10.1111/j.1467-789X.2010.00729.x
3. Bird, E. L., Baker, G., Mutrie, N., Ogilvie, D., Sahlqvist, S., & Powell, J. (2013). Behavior change techniques used to promote walking and cycling: a systematic review. Health Psychol, 32(8), 829-838. doi: 10.1037/a0032078
4. Bourke, L., Homer, K. E., Thaha, M. A., Steed, L., Rosario, D. J., Robb, K. A., . . . Taylor, S. J. (2013). Interventions for promoting habitual exercise in people living with and beyond cancer. Cochrane Database Syst Rev, 9, CD010192. doi: 10.1002/14651858.CD010192.pub2
5. Briscoe, C., & Aboud, F. (2012). Behaviour change communication targeting four health behaviours in developing countries: a review of change techniques. Soc Sci Med, 75(4), 612-621. doi: 10.1016/j.socscimed.2012.03.016
6. Cislak, A., Safron, M., Pratt, M., Gaspar, T., & Luszczynska, A. (2012). Family-related predictors of body weight and weight-related behaviours among children and adolescents: A systematic umbrella review. *Child: Care, Health and Development, 38*(3), 321-331.
7. Currie, S., Sinclair, M., Murphy, M. H., Madden, E., Dunwoody, L., & Liddle, D. (2013). Reducing the decline in physical activity during pregnancy: a systematic review of behaviour change interventions. PLoS One, 8(6), e66385. doi: 10.1371/journal.pone.0066385
8. de Young, R. (1993). Changing behavior and making it stick: The conceptualization and management of conservation behavior. *Environment and Behavior, 25*(4), 485-505.
9. Ferrier, S., Blanchard, C. M., Vallis, M., & Giacomantonio, N. (2011). Behavioural interventions to increase the physical activity of cardiac patients: a review. Eur J Cardiovasc Prev Rehabil, 18(1), 15-32. doi: 10.1097/HJR.0b013e32833ace0e
10. Golley, R. K., Hendrie, G. A., Slater, A., & Corsini, N. (2011). Interventions that involve parents to improve children's weight-related nutrition intake and activity patterns - what nutrition and activity targets and behaviour change techniques are associated with intervention effectiveness? Obes Rev, 12(2), 114-130. doi: 10.1111/j.1467-789X.2010.00745.x
11. Greaves, C. J., Sheppard, K. E., Abraham, C., Hardeman, W., Roden, M., Evans, P. H., & Schwarz, P. (2011). Systematic review of reviews of intervention components associated with increased effectiveness in dietary and physical activity interventions. BMC Public Health, 11, 119. doi: 10.1186/1471-2458-11-119
12. Heinrich, E., Schaper, N. C., & de Vries, N. K. (2010). Self-management interventions for type 2 diabetes: A systematic review. European Diabetes Nursing, 7(2), 71-76.
13. Hendrie, G. A., Brindal, E., Baird, D., & Gardner, C. (2013). Improving children's dairy food and calcium intake: can intervention work? A systematic review of the literature. Public Health Nutr, 16(2), 365-376. doi: 10.1017/s1368980012001322
14. Hendrie, G. A., Brindal, E., Corsini, N., Gardner, C., Baird, D., & Golley, R. K. (2012). Combined home and school obesity prevention interventions for children: what behavior change strategies and intervention characteristics are associated with effectiveness? Health Educ Behav, 39(2), 159-171. doi: 10.1177/1090198111420286
15. Hill, B., Richardson, B., & Skouteris, H. (2014). Do We Know How to Design Effective Health Coaching Interventions: A Systematic Review of the State of the Literature. Am J Health Promot. doi: 10.4278/ajhp.130510-LIT-238
16. Lally, P., & Gardner, B. (2013). Promoting habit formation. *Health Psychology Review, 7*(Suppl 1), S137-S158.
17. Larson, E. L., Patel, S. J., Evans, D., & Saiman, L. (2013). Feedback as a strategy to change behaviour: The devil is in the details. *Journal of Evaluation in Clinical Practice, 19*(2), 230-234.
18. Liu, S., Dunford, S. D., Leung, Y. W., Brooks, D., Thomas, S. G., Eysenbach, G., & Nolan, R. P. (2013). Reducing blood pressure with Internet-based interventions: a meta-analysis. Can J Cardiol, 29(5), 613-621. doi: 10.1016/j.cjca.2013.02.007
19. Lorencatto, F., West, R., & Michie, S. (2012). Specifying evidence-based behavior change techniques to aid smoking cessation in pregnancy. *Nicotine & Tobacco Research, 14*(9), 1019-1026. doi: 10.1093/ntr/ntr324
20. Martin, J., Chater, A., & Lorencatto, F. (2013). Effective behaviour change techniques in the prevention and management of childhood obesity. *International Journal of Obesity* (Lond), 37(10), 1287-1294. doi: 10.1038/ijo.2013.107
21. McDermott, M. S., & While, A. E. (2013). Maximizing the healthcare environment: a systematic review exploring the potential of computer technology to promote self-management of chronic illness in healthcare settings. *Patient Education and Counselling*, 92(1), 13-22. doi: 10.1016/j.pec.2013.02.014
22. Michie, S., Churchill, S., & West, R. (2011). Identifying evidence-based competences required to deliver behavioural support for smoking cessation. *Annals of Behavioural Medicine, 41*(1), 59-70. doi: 10.1007/s12160-010-9235-z
23. Michie, S., Jochelson, K., Markham, W., & Bridle, C. (2009). Low-income groups and behaviour change interventions: A review of intervention content, effectiveness and theoretical frameworks. *Journal of Epidemiology and Community Health, 63*(8), 610-622.
24. Murray, S., Sniehotta, F., Broom, J. I., Araujo-Soares, V., Dombrowski, S., & Avenell, A. (2009). Systematic review of behaviour change techniques used in interventions for weight maintenance following weight loss. *Psycholy & Health, 24,* 283-284.
25. Pal, K., Eastwood, S. V., Michie, S., Farmer, A. J., Barnard, M. L., Peacock, R., . . . Murray, E. (2013). Computer-based diabetes self-management interventions for adults with type 2 diabetes mellitus. *Cochrane Database of Systematic Reviews, 3,* CD008776. doi: 10.1002/14651858.CD008776.pub2
26. Protogerou, C., & Johnson, B. T. (2014). Factors Underlying the Success of Behavioral HIV-Prevention Interventions for Adolescents: A Meta-Review. *AIDS and Behavior*. doi: 10.1007/s10461-014-0807-y
27. Rothman, A. J., Sheeran, P., & Wood, W. (2009). Reflective and automatic processes in the initiation and maintenance of dietary change. *Annals of Behavioral Medicine*, *38(Suppl 1)*, S4-S17.
28. Tang, J., Abraham, C., Greaves, C., & Yates, T. (2014). Self-directed interventions to promote weight loss: a systematic review of reviews*. Journal of Medical Internet Research, 16*(2), e58. doi: 10.2196/jmir.2857
29. van Achterberg, T., Huisman-de Waal, G. G., Ketelaar, N. A., Oostendorp, R. A., Jacobs, J. E., & Wollersheim, H. C. (2011). How to promote healthy behaviours in patients? An overview of evidence for behaviour change techniques. *Health Promotion International, 26*(2), 148-162. doi: 10.1093/heapro/daq050
30. van der Kruk, J. J., Kortekaas, F., Lucas, C., & Jager-Wittenaar, H. (2013). Obesity: a systematic review on parental involvement in long-term European childhood weight control interventions with a nutritional focus. *Obesity Reviews*. doi: 10.1111/obr.12046
31. van Vugt, M., de Wit, M., Cleijne, W. H., & Snoek, F. J. (2013). Use of behavioral change techniques in web-based self-management programs for type 2 diabetes patients: systematic review. *Journal of Medical Internet Research, 15*(12), e279. doi: 10.2196/jmir.2800
32. Weening-Verbree, L., Huisman-de Waal, G., van Dusseldorp, L., van Achterberg, T., & Schoonhoven, L. (2013). Oral health care in older people in long term care facilities: a systematic review of implementation strategies. *International Journal of Nursing Studies, 50*(4), 569-582. doi: 10.1016/j.ijnurstu.2012.12.004
